# Physical, Nutritional, and Sensory Properties of Cheese Obtained from Goats Fed a Dietary Supplementation with Olive Leaves

**DOI:** 10.3390/ani10122238

**Published:** 2020-11-29

**Authors:** Denise Innosa, Andrea Ianni, Michele Faccia, Camillo Martino, Lisa Grotta, Maria Antonietta Saletti, Francesco Pomilio, Giuseppe Martino

**Affiliations:** 1 Faculty of Bioscience and Technology for Food, Agriculture and Environment, University of Teramo, 64100 Teramo (TE), Italy; dinnosa@unite.it (D.I.); aianni@unite.it (A.I.); lgrotta@unite.it (L.G.); 2 Department of Soil, Plant and Food Sciences, University of Bari, Via Amendola 165/A, 70126 Bari (BA), Italy; michele.faccia@uniba.it; 3 Istituto Zooprofilattico Sperimentale dell’Abruzzo e del Molise “G. Caporale”, Via Campo Boario, 64100 Teramo (TE), Italy; c.martino@izs.it (C.M.); m.saletti@izs.it (M.A.S.); f.pomilio@izs.it (F.P.)

**Keywords:** olive leaves, goat milk, linolenic acid, lipid peroxidation, volatile compounds, proteolysis

## Abstract

**Simple Summary:**

This study aims to investigate the effects of cheese obtained from goats fed a dietary supplementation with olive leaves. Thirty Saanen goats were allocated into two groups, (1) a control group that received a standard diet, and (2) an experimental group whose diet was supplemented with olive leaves. The results suggest a positive role of olive leaves in improving the cheese fatty acids composition and oxidative stability during ripening. Moreover, there were several variations in the development of volatile flavor compounds, even if no changes were evidenced in the sensory properties.

**Abstract:**

The aim of this study is to evaluate the physical, nutritional, and sensory properties of cheese obtained from goats fed a dietary supplementation with olive leaves (OL). Thirty Saanen goats were randomly allocated into two groups of 15 goats each, (1) a control group fed with a standard diet (CG), and (2) an experimental group (EG) fed an OL-enriched diet. The trial lasted for 30 days. The milk of each group was then collected and used to produce Caciotta cheese, which was analyzed at the beginning and at the end of the ripening period (60 days). The results showed a positive effect of dietary OL supplementation in improving the fatty acid profiles due to the significant increase of unsaturated fatty acids, mostly α-linolenic acid (C18:3 n-3), with the consequent reduction of the ω-6/ω-3 ratio, a condition commonly associated with an increased health functionality of food products. Moreover, improved oxidative stability was observed in cheese during ripening, a presumable consequence of the transfer into the milk of dietary bioactive compounds, mainly polyphenols of high biological value, and credited as a marked antioxidant potential. Furthermore, reduced lipolytic action was observed in 60-day ripened cheese, even if no significant changes in sensory properties were evidenced.

## 1. Introduction

In recent years, several studies have focused on the importance of the recovery of by-products derived from the olive oil extraction process and olive tree pruning. The term “olive leaves” (OL) refers to a mix of leaves and branches obtained from olive tree pruning and olive harvesting and cleaning [1]. Such a mix appears to be a copious by-product, representing (approximately) 10% of the total weight of harvested olives, and reaching an average of 25 kg per olive tree during the pruning period [2]. In light of the above, OL disposal often represents a non-negligible problem for companies operating in the sector, from an environmental and an economic point of view.

Nowadays, OL find different applications in pharmaceutical and cosmetic fields, as well as in the food sector, as it is useful in the production of additives, credited to prominent antioxidant, anti-inflammatory, and antimicrobial activity [3,4]. These industrial applications spread mainly as a consequence of the high biological value of this by-product, which is due to the presence of well-known and characterized phenolic bioactive compounds, such as caffeic acid, tyrosol, hydroxytyrosol, flavones (apigenin, kaempferol, and luteolin), and oleuropeosides (oleuropein and verbascoside) [5].

Considering this premise, it is plausible that OL can be used as a resource in the zootechnical field, as a dietary supplement for farm animals, by (1) identifying eco-sustainable ways of using OL, (2) enriching the diet of farm animals with compounds potentially useful for preserving animal welfare, and (3) improving qualitative and quantitative characteristics of production.

Recent studies show how OL can induce positive effects when integrated in animal diets. For instance, a study by Cayan and Erener [6] measured the effects of dried OL powder on the performance of laying hens. Their results showed a reduction in the egg yolk cholesterol content. Positive effects of OL supplementation were also demonstrated by Botsoglou et al. [7], who observed an improvement of pork meat quality because of the inhibition of lipid oxidation by antioxidants contained in the by-product. Regarding the use of this vegetable matrix in goats, a similar study conducted by Innosa et al. [8] should be noted; the authors observed an improvement in the fatty acid composition and oxidative stability of Ricotta cheese—a fresh cheese that is generally consumed within a few days from production.

However, there is a lack of similar studies on ripened goat dairy products. Thus, this study focuses its attention on the properties of 60-day aged goat’s milk cheese, Caciotta cheese, from goats fed a dietary supplementation with OL. Moreover, a further purpose of the investigation was to identify a breeding strategy able to induce improvement in the qualitative characteristics of the product, for which there has been increasing interest in the last few years. Indeed, for some time now, goat’s milk and cheese have been considered excellent alternatives to cow’s milk products, as goat’s milk products are able to satisfy a wide range of individual nutritional needs, specific to age, lifestyle, physical activity, and one’s state of health. Furthermore, goat’s milk is generally better tolerated; it stands out as it is easier to digest (the fat globules are smaller), it is a good source of calcium and phosphorus, is rich in vitamins, and optimal for bone mineralization. Finally, goat’s milk is the ruminant milk that is most similar to breast milk; thus, it is ideal for children [9,10,11].

## 2. Materials and Methods

### 2.1. Experimental Design, Cheesemaking, and Sampling

The experimental plan was performed according to Directive 2010/63/EU of the European Parliament (European Union, 2010) and Directive 86/609/EEC (European Economic Community, 1986), which deal with the protection of animals used for scientific purposes [12,13]. The trial was conducted in a commercial company that, during the spring months, normally introduces (into the diet of lactating goats) residues coming from olive tree pruning. For this reason, no breeding practices other than those normally adopted have been introduced; therefore, approval by the ethics committee was not considered necessary.

Thirty Saanen goats, homogeneous for age (46 ± 2 months), weight (52.7 ± 4.3 kg), lactation days (86 ± 7 days in milk), milk yield (2296 ± 281 g/day), and body condition (BCS 2.78 ± 0.18) were allocated into two groups of 15 goats each, (1) a control group (CG), and (2) an experimental group (EG), whose diets were enriched with olive leaves. The trial period lasted 30 days. For the entire time, the goats were kept in two adjacent (but separate) areas; in each area, it was possible to obtain a space shared by animals belonging to the same group, a drinking trough, and provisional single bunks on straw, useful for the individual administration of the diet. The administered diets were formulated to be isoproteic and isoenergetic. Every day, each group of animals received polyphite hay, administered *ad libitum*, twice daily, in the morning (08:00 h) and in the evening (18:00 h). Animals individually received a custom-formulated concentrate (Table 1) for a total of 1 kg/head; for EG goats, the concentrate was supplemented with OL (approximately 10% on a dry matter (DM) of the whole diet). The use of the previously mentioned individual bunks was helpful for monitoring the amount of daily-ingested feed. Twice a week, the daily milk production was evaluated.

Feed samples were analyzed for DM (method 930.15), crude protein (CP; method 954.01), ether extract (EE; method 920.39), crude fiber (CF; method 962.09), and ash (method 942.05), according to the methods of analysis of the Association of Official Analytical Chemists (AOAC, 1990) [14]. Neutral detergent fiber (NDF) and acid detergent fiber (ADF) were determined by the detergent procedures of Goering and Van Soest [15]. Table 1 also shows the fatty acid composition of OL and diets administered to the CG and EG, evaluated according to the procedure reported by Castellani et al. [16].

At the end of the trial, individual milk samples were collected and used for the evaluation of chemical properties and milk fatty acid profiles. The remaining milk of each group was then pooled and used for cheese production by following the manufacturing protocol reported below. Bulk milk was pasteurized at 72 °C for 20 s, and cooled to 40 °C ± 1 °C. Then, the milk was transferred in a container in which a combination of thermophilic and mesophilic starter culture was added (500 U/5000 L; White Daily, Chr Hansen, Hoersholm, Denmark). After acidification, rennet was added to the milk at the ratio of 1:20,000 (75% of chymosin and 25% of pepsin; Clerici, Cadorago, Italy). Thus, 45 min of incubation was needed; then, the curd was broken into small pieces resembling the size of hazelnuts, and portioned in aliquots of ca. 1 kg, in plastic molds, and kept at 48 °C ± 1.5 °C until the pH reached at 5.20 ± 0.2. At this time, all the forms were salted with a solution of NaCl 20% in water, and then stored in the ripening room with controlled temperature and humidity (10 °C ± 0.5 °C; 85%, respectively). To evaluate changes in the chemical and nutritional parameters through the ripening time, from the cheesemaking process, six wheels of cheese per group were prepared. Three wheels were sampled after 1 d of ripening (T1), and the other three after 60 d of ripening (T60).

### 2.2. Chemical Composition of Milk and Cheese

The chemical composition of milk was determined on individual milk samples collected at the end of the trial. Fat, protein, casein, lactose, and urea contents were determined using a MilkoScan FT 6000 (Foss Integrator IMT: Foss Analytics, Hillerǿd, Denmark). The milk lipid fraction was extracted following the AOAC official method [14].

Regarding cheese, DM and total proteins were determined according to AOAC methods [17]. The lipid extraction was carried out via acid hydrolysis. Briefly, 3 g of cheese were homogenized with 10 mL of HCl 25% in water and, after deproteinization, lipids were extracted with 90 mL of diethyl ether and petroleum ether (1:1).

### 2.3. Cheese Color Measurement

The color of Caciotta cheese at two different ripening times was performed with the colorimeter Konica Minolta Chroma Meter CR-5, according to the International Commission on Illumination (Commission Internationale de L’éclairage (CIE), 1978). Cheese forms were cut in half and color measurements always took place on the inner surfaces of each half of cheese form. Color parameters were determined for the edge part and for the middle part of the samples, and measurements were performed in triplicate. The size of the aperture of the optical system was adjusted to 3 mm and the differences between samples were evaluated in individual parameters as L* (lightness), a* (green-red value), and b* (blue-yellow value), and by using the total color differences (ΔE*ab), and the yellow index (YI), calculated using the following formulas:

ΔE*_ab_ = [(ΔL*)^2^ + (Δa*)^2^ + (Δb*)^2^]^1/2^ (1)

YI = 142.86 × b*/L*(2)

### 2.4. Fatty Acids Profile of Milk and Cheese and Lipid Peroxidation

For both milk and cheese, 50 mg of extracted lipids were weighed and reconstituted with 1 mL of hexane containing C21:0 methyl ester as internal standard (Sigma Aldrich, Milan, Italy) and methylated with 500 µL of 2N sodium methoxide solution in methanol [18]. Detection of fatty acids methyl esters (FAME) was performed through the use of gas chromatography (GC) coupled with a flame ionization detector (FID) and equipped with a capillary column (Restek Rt-2560 Column, fused silica 100 m × 0.25 mm highly polar phase; Restek Corporation, Bellefonte, PA, USA). Hydrogen was used as a carrier gas at a flow rate of 1 mL/min, and the temperatures of the injector and the detector were set up at 280 °C. The initial temperature was held at 80 °C for 10 min; then it was increased to 172 °C at 4 °C/min, held for 30 min, and finally, increased to 190 °C at 4 °C/min, and held for 10 min, for a total run time of 56 min. Identification of each FAME was made by comparing the retention time of each compound with the retention time of a mix of FAME analytical standards (F.A.M.E. Mix C8-C24; Supelco, Bellefonte, PA, USA). Peak areas were quantified using Chrome Card Software and the results for each fatty acid (FA) were expressed as a mean percentage of a single compound of the total FAME. The relative percentage of each fatty acid was also used to calculate atherogenic (AI), thrombogenic (TI), and the desaturation indices (DI), as previously reported [8].

The evaluation of lipid peroxidation in samples of freshly prepared and ripened cheese was performed through the Thiobarbituric Acid Reactive Species (TBARS) test, according to the procedure described by Ianni et al. [19]. The results were expressed in µg equivalent of malondialdehyde (MDA) per gram of cheese.

### 2.5. Cheese Volatile Profile

Volatile compounds (VOCs) were extracted via solid-phase microextraction (SPME) and the separation, and later, identification was performed with a gas chromatography (GC Clarus 580; Perkin Elmer, Waltham, MA) coupled with a mass spectrometry (MS SQ8S; Perkin Elmer), in according with Ianni et al. [20]. The GC-MS was equipped with an Elite-5ms column (length: 30 m; internal diameter: 0.25 mm; film thickness: 0.25 μm; Perkin Elmer, Waltham, MA, USA), and helium was used as carrier gas at a flow rate of 1 mL/min. The oven temperature was initially settled at 50 °C and held for 1 min, and then increased up to 200 °C with a ratio of 3 °C/min, held for 1 min, and finally increased again to 250 °C, with a ratio of 3 °C/min and held for 15 min. The mass spectrometer operates in electronic impact ionization mode a t 70 eV. About 3 g of Caciotta cheese were transferred in vials and mixed with 10 mL of NaCl solution (360 g/L) and with 10 µL of 4-methyl, 2-heptanone was used as internal standard. The sealed vial with the sample was stirred at 60 °C for 60 min in a thermostatic bath to allow the absorption of the VOCs to the divinylbenzene-carboxen-polydimethylsiloxane SPME fiber in the headspace. Then, VOCs extracted were thermally desorbed in the GC injector for 1 min, in a splitless mode at 250 °C. Volatile compounds were identified by comparison with mass spectra included in the library database (NIST Mass Spectral library, Search Program version 2.0, National Institute of Standards and Technology, U.S. Department of Commerce, Gaithersburg, MD, USA), and by comparing the elution order with Kovats indices. Analysis were performed in triplicate and data were expressed as relative abundance, as a percentage of each compound on the sum of the total VOCs.

### 2.6. Evaluation of Cheese Electrophoretic Profile

Protein degradation was evaluated through SDS-PAGE in accordance with the Laemmli procedure [21]. Briefly, 1 g of Caciotta cheese was homogenized with 20 mL of tris-glycine 0.01 M, pH 8.3 and UREA 6 M. This was incubated at 37 °C for 40 min to facilitate casein solubilization. After cooled to room temperature, the extract was centrifuged at 10,000× g, 4 °C for 15 min. Then, supernatant was recovered and filtered with Whatman filter paper to remove fat and other insoluble solids. Proteins were quantified using the Bradford method [22], using BSA as standard. Protein extract was mixed with an equal volume of sample buffer (0.5 M tris-HCl pH 6.8, 2% *w/v* SDS, 7% *v/v* glycerol, 4.3% *v/v* β-mercaptoethanol, 0.0025% *w/v* bromophenol blue), and the mixture was boiled for 5 min to inactivate enzymes and denature proteins. Moreover, 10 µg of protein samples were loaded onto a 15% SDS-PAGE for separation. Gels were stained with Coomassie Blue R-250 solution 0.5 *w/v* solution dissolved in 50% methanol and 10% acetic acid for 45 min, and destained with 50% methanol and 10% acetic acid. Polypeptides molecular weights were estimated using a molecular weight calibration kit (Precision Plus Protein All Blue Standards, Bio-Rad, Segrate, Italy) and SDS-PAGE was performed in triplicate. Densitometric analysis of bands was performed with ImageJ Software [23] and the content of caseins and low molecular weight products (LMWP) was expressed as a percentage of the total protein content.

### 2.7. Evaluation of Cheese Texture Profile and Sensory Analysis

Texture profile analysis allowed evaluating the mechanical properties of the cheese. Analyses were performed with the dynamometer Instron UTM 5542 (Instron, Wycombe, UK), equipped with a flat probe of a 3 cm diameter. The instrumentation allows reproducing the conditions applied during mastication through a double cycle compression test (Texture Profile Analysis, TPA; Bourne, 1968). The parameters evaluated were hardness (N), cohesiveness, gumminess (N), and the young module (N/mm^2^). A pre-load of 0.05 N, a test speed of 30 mm/min, and a deformation of 30% were adopted as experimental parameters for all of the analyses. For each cheese sample, a minimum of 20 parallelepiped-shaped aliquots (1.5 × 1 × 1 cm) were prepared, equilibrated at 20 °C, and analyzed. Regarding sensory analysis, a “double step” approach was used. The first step was a “multiple” paired comparison test to assess the presence of differences between samples as to appearance, odor, and taste. The sample pair (control and experimental) was presented to each panelist two times in a balanced way (AB–BA or BA–AB), in white disposable dishes labeled with three digit codes. At the first presentation of the pair, the panelists were asked only to observe the samples and answer the question: (a) “Are the samples different?”. At the second presentation, the panelists were asked to sniff and taste the samples, then to answer the questions: (b) “Are the samples different in odor?”; (c) “Are the samples different in taste ?”. The analyses were done in duplicate (two different sessions separated by 1 h). The second step was a quantitative descriptive analysis applied to describe and quantify the differences found during the paired comparison test. In this case, the two samples were presented one at time to the panelists, who were asked to score them using a 0–5 hedonic scale, only for the discriminating characteristics arisen during the paired comparison tests. Among them, the texture attributes were chosen from the descriptors list included in the Italian Association of Cheese Tasters (ONAF) vocabulary [24]. All analyses were carried out by a panel composed of 12 assessors (7 male and 5 female, aged 27–62 years), selected and specifically trained according to International Organization for Standardization (ISO) 22935-1:2009 [25], from a group of 33 people belonging to the Italian Association of Cheese Tasters (ONAF), who had followed a 20 h course on evaluation of appearance, texture, odor, and taste of cheese.

### 2.8. Statistical Analysis

Analyses were performed in triplicate and the results were reported as mean ± standard deviation. Regarding, the analysis of statistically significant differences between the two groups of data, the SigmaPlot 12.0 Software (Systat software Inc., San Jose (CA), USA) for the Windows operating system was used (ANOVA, Student’s *t* test); differences with a *p* value lower than 0.05 were considered statistically significant.

## 3. Results

### 3.1. Milk and Cheese Chemical Composition and Caciotta Cheese Color

In individual milk samples, as reported in Table 2, the dietary OL integration did not induce significant variations on the casein, lactose, fat, total protein, and urea contents (*p* > 0.05). Regarding the cheese chemical composition (Table 3), the moisture was significantly lower in the EG samples compared with the CG samples (*p* < 0.05) at T60.

The diet enriched with OL also affected the color parameters in Caciotta cheese. Lightness (L*) was lower in the EG cheeses, both at T1 and T60 (*p* < 0.01 both at T1 and T60). The redness (a*) was not affected by the feeding strategy, while yellowness (b*) was significantly lower in the EG samples compared with the CG samples after 1 and 60 days of ripening (*p* < 0.05 and *p* < 0.01 for T1 and T60 samples, respectively). The yellow index (YI) was not significantly affected by the diet.

### 3.2. Fatty Acids Profile and Lipid Peroxidation

The diet enriched with OL affected the fatty acids profile of milk (Table 4). The major changes concerned the increase of stearic (C18:0; *p* < 0.05), vaccenic (C18:1 *trans*-11, *p* < 0.05), oleic (C18:1 cis-9, *p* < 0.01), and linolenic (C18:3, *p* < 0.05) acids, and a decrease of palmitic acid (C16:0, *p* < 0.05). Furthermore, it was possible to observe a decrease of the ratio omega (ω)-6/ omega (ω)-3 (*p* < 0.001). As observed in milk, the fatty acids profile of the cheese was influenced by the diet (Table 4). In particular, major changes were observed regarding the increase of vaccenic (C18:1 *trans*-11, *p* < 0.01) and linolenic (C18:3, *p* < 0.01) acids, and the decrease of the ratio ω-6/ω-3 (*p* < 0.001).

**Figure 1:**
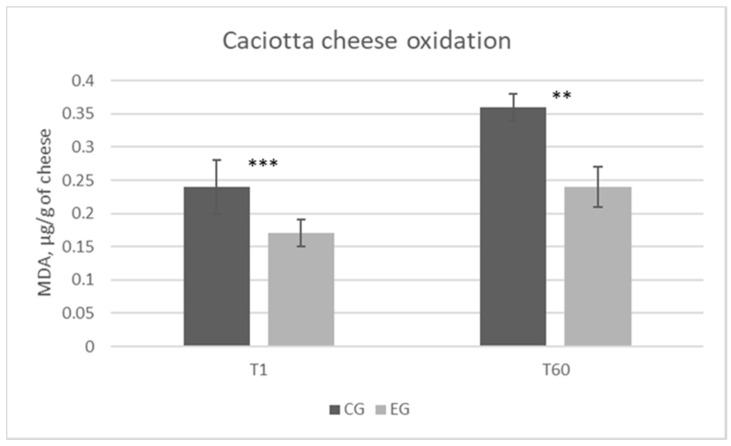
Lipid peroxidation profile of cheese samples obtained from the control group (CG) and the experimental group (EG). Analysis performed on samples obtained after 1 (T1) and 60 (T60) d of ripening. MDA = malondialdehyde; ** *p* < 0.01; *** *p* < 0.001.

The lipid oxidative process was determined by the TBARS-test after 1 day (T1) and 60 days (T60) of ripening. As shown in Figure 1, at the time of ripening (T1 and T60), the total amount of MDA was significantly lower in the EG compared with the CG samples (*p* < 0.001 and *p* < 0.01 at T1 and T60, respectively).

### 3.3. Volatile Compounds

Dietary integration with olive leaves significantly affected the aromatic profile of Caciotta cheese analyzed after 1 (T1) and 60 (T60) days of ripening. As reported in Table 5, in all of the samples were found 45 compounds: 9 alcohols, 11 aldehydes, 8 carboxylic acids, 5 ethyl esters, 4 ketones, 5 lactones, and 3 other compounds. In fresh cheese (T1), only alcohols and the other compounds were significantly different between the CG and EG samples. In fact, both alcohols and other compounds were higher in the EG Caciotta compared with the CG Caciotta (*p* < 0.05 for both alcohols and other compounds). After 60 days of ripening, in cheeses obtained by feeding goats with the OL supplementation, it was possible to observe an increase of ethyl esters (*p* < 0.05) and ketones (*p* < 0.01), and a decrease of alcohols (*p* < 0.05), carboxylic acids (*p* < 0.05), and lactones (*p* < 0.05). After 1 day of ripening, the only alcohol higher in the EG samples compared with the CG samples was 1-hexanol, 2-ethyl (*p* < 0.05). After 60 days of ripening, 1-hexanol, 2-ethyl (*p* < 0.01) and 1-heptanol (*p* < 0.01) were lower in the EG samples compared with the CG samples, while 1-nonanol (*p* < 0.001) and 2-nonanol (*p* < 0.05) were higher. Among the aldehyde compounds, at T1 solely 2-octenal (*p* < 0.05) appear to be significantly higher in EG cheese compared with CG cheese; at T60, the aldehyde found lower were nonanal (*p* < 0.05), benzaldehyde (*p* < 0.05), and benzaldehyde, 3-ethyl (*p* < 0.05). Concerning the carboxylic acids, in fresh cheese (T1), octanoic acid (*p* < 0.05) were higher in the EG Caciotta compared with the CG Caciotta, while dodecanoic acid (*p* < 0.05) was lower. After 60 days of ripening, almost all of the carboxylic acids were lower in the EG samples compared with the CG samples: butanoic acid (*p* < 0.05), hexanoic acid (*p* < 0.05), octanoic acid (*p* < 0.05), nonanoic acid (*p* < 0.01), decanoic acid (*p* < 0.05), and dodecanoic acid (*p* < 0.01). Regarding the ethyl esters compounds, no significant differences were found after 1 day of ripening, while sundry ethyl esters were higher after 60 days of ripening in cheeses obtained from goats fed with olive leaves: hexanoic acid ethyl ester (*p* < 0.05), octanoic acid ethyl ester (*p* < 0.05), decanoic acid ethyl ester (*p* < 0.05). Concerning the ketones, in T1 samples for the EG cheese, it was possible to observe an increase of 2-nonanone (*p* < 0.05) and a decrease of acetoin (*p* < 0.05); in T60 samples, an increase of 2-heptanone (*p* < 0.05) and 2-nonanone (*p* < 0.05) was observed. With regard to lactones, δ-decalactone resulted significantly lower both in T1 EG samples (*p* < 0.05) and in samples obtained after 60 days of ripening (*p* < 0.05); δ-dodecalactone (*p* < 0.05) was lower only in the EG samples after 60 days of ripening while ε-dodecalactone (*p* < 0.01) was not significantly affected from the diet. Finally, ethylbenzene (*p* < 0.001) and caryophyllene (*p* < 0.05) were found to be significantly higher in the EG fresh cheese.

### 3.4. SDS-Page Analysis

**Figure 2:**
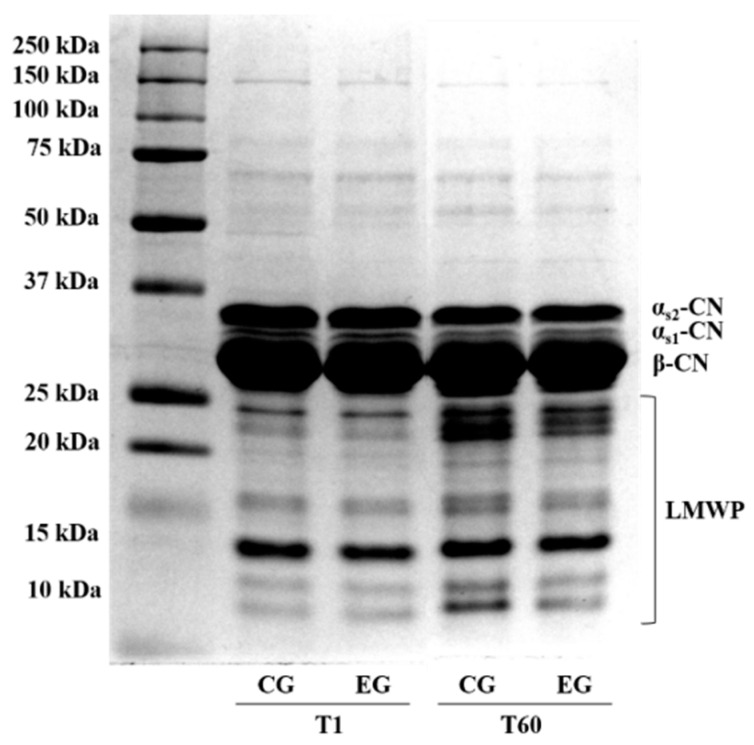
Results of SDS-PAGE analysis of protein in cheeses at 1 (T1) and 60 (T60) d of ripening from goats fed a standard diet (CG) or diet supplemented with olive leaves (EG). α-CN: α-casein; β-CN: β-casein; LMWP: low molecular weight protein.

The degradation of caseins extracted from fresh (T1) and ripened (T60) cheeses was evaluated through sodium dodecyl sulfate polyacrylamide gel electrophoresis (SDS-PAGE) analysis. As it is possible to observe in Figure 2, cheese proteins were separated into clear bands that can be identified αs_2_-casein (αs_2_-CN), αs_1_-casein (αs_1_-CN), β-casein (β-CN), whereas the bands having lower molecular weight (LMWP) should be referred to casein fragments and traces of whey proteins (β-lactoglobulin and α-lactalbumin).

Under the applied experimental condition, it was possible to observe in all CG and EG samples, at T1 and T60, a major β-CN, and less intensive bands for α-casein isoforms (αs_2_-CN and αs_1_-CN). The diet enriched with olive leaves used to feed the experimental group goats did not seem to influence the protein profile of both fresh (T1) and ripened (T60) cheeses (Table 6).

### 3.5. Cheese Texture and Sensory Evaluation

The texture profile analysis showed significant differences between T60 samples (Table 7). No significant differences were observed for samples of fresh cheese, while after 60 days of ripening, the EG cheese was harder (*p* < 0.001), more rubbery (*p* < 0.001), and more elastic (*p* < 0.001) compared to the CG cheese.

**Figure 3:**
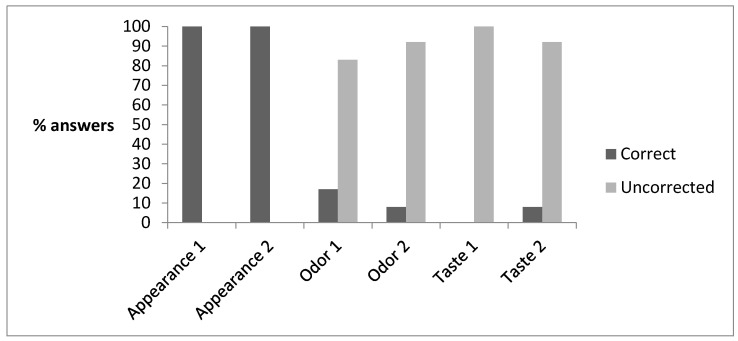
Results of the paired comparison tests in terms of % of correct and uncorrected answers (2 replicates) in the discrimination of samples with regard to the parameters reported on the abscissa axis (appearance, odor, and taste). The dark colored columns represent the percentage of correct answers, while the percentage of uncorrected answers is represented by light colored bars.

The results of the paired comparison test are shown in Figure 3, whereas those obtained from Quantitative Descriptive Analysis (QDA) are reported in Table 8. The panelists almost unanimously discriminated the control and experimental samples by appearance, whereas they judged them as not different in odor and taste. The reasons for the differences found in the appearance were clarified by the QDA analysis. The only attribute that was significantly different under the statistic point of view was “eyes”, even though color approached the 0.05 *p-*value of significance (0.077). This latter result matched well with those obtained by instrumental analysis.

## 4. Discussion

The dietary OL supplementation did not influence the chemical–nutritional composition of milk, or the milk production, for the entire duration of the trial. This finding is, therefore, indicative of the fact that the experimental feeding strategy had no effect on the functionality of the mammary gland, which is closely related to the secretion of fat, caseins, and total protein in milk [26].

With regard to the chemical composition of cheese, no differences in the content of total fat were highlighted, while the color evaluation evidenced a significant reduction in lightness in the EG samples, at the end of the ripening period (T60). As previously reported by El-Nimr et al. [27], the lightness in a dairy product can be directly related to the moisture content. Since our study has shown a greater loss of humidity by the EG cheese after 60 days from the cheesemaking, it is plausible that this could represent the most obvious key reading of the observed finding. In addition, the EG samples, both after 1 and 60 days of ripening, are characterized by significantly lower values of the chromatic coordinate b*, which testifies to the tendency of the dairy product to take on a darker totality. This finding can be partially explained by taking into account the mentioned lower presence of water in the EG cheeses. However, there are references in the literature that attribute this type of variation to the presence of water-soluble or fat-soluble metabolites that can be taken by ruminants with the diet, and released into the milk through the mammary gland. For instance, the cheese content of fat-soluble vitamins, such as retinol and xanthophyll, are responsible for higher values of the b* parameter, which entails a tendency for the cheese to take on a yellowish and, therefore, lighter color [28]. Considering this, it is plausible to hypothesize a shortage of these compounds in the diet supplemented with OL; however, it is not possible to make specific assumptions on this aspect, as no analyzations aimed at ascertaining this have been conducted. Lastly, considering the total color difference (ΔE*ab), it is possible to assert that there is a middle difference between CG and EG cheeses after 1 and 60 days of ripening time [29].

The dietary supplementation with OL significantly affected the fatty acid composition of both milk and cheese. Relatively, to milk, it was possible to highlight a significant increase in concentration of stearic (C18:0), oleic (C18:1 *cis*-9), vaccenic (C18:1 *trans*-11), and linolenic (C18:3 *cis*-9, *cis*-12, *cis*-15) acids, while in cheese, the behavior was confirmed only for vaccenic and linolenic acids. In order to justify these findings, it is useful to refer to the evaluation of the fatty acids profile in standard and experimental diets (Table 1). In fact, OL integration induced a significant increase in concentration of stearic and linolenic acids, while a significant reduction of linoleic acid was detected in the ration administered to the EG goats. As previously demonstrated, the linolenic acid taken from the diet by ruminants represents a useful substrate for ruminal biohydrogenation processes, with the consequent production of vaccenic and stearic acid following enzymatic events of isomerization and reduction. In turn, these fatty acids, after leaving the ruminal environment, are captured by other tissues, with a consequent increase in their concentration in milk and meat. In addition, vaccenic and stearic acid also represent the precursors of other important endogenously synthesized fatty acids, moreover, conjugates of linoleic acid (CLA). In this case, reference is made to metabolic events mediated by Δ^9^-desaturase, which convert stearic acid into oleic acid, and vaccenic acid (C18:1 *trans*-11) into rumenic acid (C18:2 *cis*-9, *trans*-11) [30]; thus, contributing to the increase in the concentration of these fatty acids in products of animal origin. In this study, however, it should be pointed out that, in both milk and cheese, no differences were highlighted relating to rumenic acid and, more generally, in the sum of the (CLA); an aspect that implies metabolic events that should be appropriately characterized in a more specific way. The higher amount of linolenic acid in both milk and cheese is also responsible for a significant decrease of the ω-6/ω-3 ratio. This condition is generally associated with an improvement in the health properties of the food products, as it correlates with a reduction of risks for cancer growth and development of coronary heart disease [31].

Regarding the oxidation status of fresh and ripened cheeses, the evaluation was performed through the TBARS-test. The diet enriched with OL positively affected the oxidative stability of cheese samples after 1 day (T1) and 60 days (T60) of ripening. Generally, the tendency of a food to oxidize strongly depends on the Polyunsaturated Fatty Acids (PUFA) concentration. The well-known and characterized tendency of PUFA to oxidize is therefore a phenomenon of great importance for the food sector, as a consequence of the fact that foods rich in these compounds can undergo deterioration with detrimental effects on both nutritional quality and food safety [32]. In this study, despite the significant increase in concentration of linolenic acid in EG samples, no changes were observed in the overall sum of PUFA. The greater resistance to lipid peroxidation showed by the EG cheeses was also found in other studies, in which the diet of lactating ruminants was integrated with plant matrices. The most common and plausible explanation of the phenomenon concerns the fact that these matrices are generally rich in bioactive compounds, mainly polyphenols, of high biological value, and credited for their potential antioxidant activity [33].

Regarding the evaluation of volatile profile, it is important to underline the fact that, in recent years, numerous studies on ruminants highlighted the influence of the feeding strategy to induce variations in the release of VOCs in milk and derived dairy products [34]. In our study, free fatty acids (FFAs) represent the most abundant class of compounds found in fresh (T1) and ripened (T60) samples, testifying for the prevalence of the lipolytic processes compared to the proteolytic events. Taking into consideration the comparison between the cheese samples derived from the two experimental groups, the greatest differences were highlighted, as expected, at the end of the ripening period (T60 samples). The T60 EG samples evidenced lower concentrations of FFAs, a finding specifically due to the reduction in the release of butanoic, hexanoic, octanoic, nonanoic, decanoic, and dodecanoic acids. A similar behavior was recently reported by Bennato et al. [35], who evaluated the volatile profile in goat-ripened cheeses obtained by enriching the animal diet with dried licorice root. In that case, authors justified this phenomenon by supposing a role of plant matrix bioactive compounds to slow down the lipolytic mechanisms during the ripening period. Of particular interest for our study is the finding related to the reduction in concentration of hexanoic, octanoic, and decanoic acids in the EG samples. These compounds are, in fact, commonly associated to strong and unpleasant odors, defined as sweaty, rancid, and cheesy [36]; therefore, their excessive production could have undesirable effects in the determination of the aroma and flavor of the dairy products.

The FFA catabolism was also reported to be responsible for the release of compounds capable of influencing cheese aroma; these compounds mostly belong to the families of straight-chain aldehydes, secondary alcohols, ethyl esters, methyl ketones, and lactones [37].

Another important group of VOCs in this study was represented by aldehydes; these compounds generally derive from the non-enzymatic intrachain oxidation of unsaturated fatty acids characterizing the cheese matrix, causing the release of hydroperoxides, which are rapidly converted in aldehydes (mainly from C5 to C9), whose accumulation in food matrices is generally responsible for the off-flavor development [37,38]. In this study, no significant differences were found in the sum of these compounds in both T1 and T60 samples. The greatest concentration of aldehydes was specifically found in fresh cheeses (in both the CG and EG samples) with a significant reduction during ripening. Such phenomenon testifies to the onset of biochemical mechanisms that may have converted these compounds into other chemical forms. In this regard, a possible mechanism concerns the reduction of aldehydes to form primary alcohols, such as 1-hexanol, 1-heptanol, 1-octanol, and 1-nonanol, which did not show particular variations in the analyses conducted in this study of both fresh and aged cheeses.

FFAs can undergo oxidation and generate β-ketoacids, which can be quickly converted to the corresponding methyl ketones through a decarboxylation mechanism. Methyl ketones, especially 2-heptanone and 2-nonanone, are reported to be responsible for the development in dairy products of a characteristic flavor, defined as fruity, spicy, and musty [39]. The considerable increase in concentration of these compounds in the EG T60 samples should therefore be able to justify a sensitive variation on a sensory level.

Furthermore, it should be highlighted that the increase of ethyl esters and the contemporary decrease in concentration of both alcohols and FFAs in EG samples after 60 days of ripening, could suggest that the synthesis of ethyl esters presumably occurred by enzymatic esterification of alcohols and carboxylic acids. Ethyl esters are generally responsible for pleasant, fruity notes that reduce cheese sharpness and bitterness. In our EG samples, the most representative and significantly higher ethyl esters were ethyl hexanoate, ethyl octanoate, and ethyl decanoate, which accumulated only in cheeses analyzed at the end of the ripening period.

Finally, interesting results were obtained from the identification of lactones. These VOCs can be released from hydroxylated FFAs by enzymatic reaction or induced by a heating process [40]. Lactones, such as δ-decalactone and δ-dodecalactone, which resulted significantly lower in EG samples, are reported to be associated with very pronounced fruity notes, although they have been found to also contribute to cheese (the buttery character) [41].

By focusing the attention on VOCs derived from proteolytic events, phenylacetaldehyde, 2-phenylethylalcohol, and 3-methyl-1-butanol were identified in both the CG and EG cheese. The first two compounds derive from the phenylalanine catabolism, while the last is obtained from the leucine catabolism [42]. The 2-phenylethylalcohol and 3-methyl, 1-butanol, were observed only in the CG and EG samples at the end of the 60 days of the ripening period, while phenylacetaldehyde also resulted in fresh CG and EG cheese samples. However, the diet did not involve any significant differences for these compounds.

The inability of feed supplementation to induce significant variations in the proteolytic processes was also highlighted through the SDS-PAGE analysis, which was specifically useful in the evaluation of primary proteolysis in cheese samples. The electrophoretic profile in all samples was characterized by caseins (αs2-CN, αs1-CN, and β-CN) and eight different low molecular weight proteins (LMWP, from 25 kDa to 10 kDa), some of which should be products arising from the proteolytic activity. The dietary supplementation with OL did not generate significant differences for αs2-, αs1-, β-CN, or the LMWP. The only finding that emerges from this analysis concerned the occurrence of a slight proteolytic process in both CG and EG samples as an effect of ripening, as evidenced by the reduction in all T60 samples of the relative amount of caseins, and a concomitant increase in the presence of some protein breakdown products. This result was also confirmed by the small amounts of soluble nitrogen detected in T60 samples by Kjeldahl, and HPLC analyses (results not shown). A further aspect that clearly appeared from the SDS-PAGE was that the milk used contained a low level of αs1-CN. The scarce presence of this protein fraction in milk can partially explain the low level of proteolysis observed in cheese, since it is known that αs1-CN is the most degraded casein, whereas the β fraction is relatively resistant to enzyme hydrolysis [43]. A second aspect to consider, in order to justify the observed mild proteolysis, is the fast decrease in moisture (much less than 40% at day 60 of ripening), which is typical in small-sized hard cheeses.

Textural profile analysis allowed evaluating the physical properties of cheese, which are commonly able to differentiate between many cheese varieties, and are considered by the consumer as determinants of overall quality and preference [44]. The differences usually observed in textural properties of ripened cheese could be totally attributed to variations in the moisture content; the water is in fact able to act in dairy products as a plasticizer, inducing a liquid-like behavior [45]. As expected, lower moisture content was detected in ripened EG samples (T60), resulting in a harder texture characterized by difficultly to break structural interactions [46]. However, this finding, in association with what was observed for gumminess, and the Young Module, should be characterized in more detail, presumably considering the presence in cheese of specific organic compounds directly derived from the OL-supplemented diet.

Regarding the results obtained with sensory analysis, it can be concluded that the feeding treatment had no direct effect on the sensory characteristics of the cheeses. Nevertheless, the main difference observed (i.e., higher presence of eyes in the untreated sample) is worth studying, since it can be a random result or derive from an antimicrobial effect connected to the feeding treatment.

## 5. Conclusions

The results obtained in this study suggest the positive role of dietary supplementation with OL on the nutritional characteristics of goat’s milk and its derived dairy products. In particular, it was possible to observe a better oxidative stability, in both freshly prepared and ripened cheese samples. Furthermore, cheese obtained from goats fed the OL dietary supplementation was characterized by lower lipolytic action due to the presumably positive role of plant matrix bioactive compounds to slow down the lipolytic mechanisms during the ripening period. In addition, the highlighted variations in the volatile profile did not lead to sensory alterations. Therefore, the dietary OL supplementation did not influence consumer acceptability of the obtained dairy product.

## Figures and Tables

**Table 1:** Ingredients, chemical composition of fatty acid profile of diets administered to the control group (CG) and the experimental group (EG) that received the dietary olive leaves (OL) supplementation.

**Chemical composition of polyphite hay**			
Dry Matter (DM)		87.50	
Ash ^1^, %		10.29	
Crude Protein (CP) ^1^, %		12.80	
Ether Extract (EE) ^1^, %		2.86	
Neutral Detergent Fiber (NDF) ^1^, %		59.66	
Acid Detergent Fiber (ADF) ^1^, %		31.89	
**Ingredients of the concentrate (%)**		**CG**	**EG**
Corn, meal		25	25
Soy flour (44% crude protein)		23	23
Barley, meal		20.8	20.8
Calcium carbonate		0.4	0.4
Dicalcium phosphate		0.3	0.3
Beet pulps		30	-
Olive leaves (OL)		-	30
Vitamins		0.5	0.5
**Chemical composition of the concentrate (%)**	**OL**	**CG**	**EG**
Dry Matter (DM)	90.5	88.50	90.01
Ash ^1^, %	4.70	5.08	6.39
Ether Extract (EE) ^1^, %	2.90	2.40	2.66
Crude Protein (CP) ^1^, %	8.50	18.73	18.04
Neutral Detergent Fiber (NDF) ^1^, %	37.40	21.89	20.84
Acid Detergent Fiber (ADF) ^1^, %	29.60	11.67	12.76
**Fatty Acids ^2^**	**OL**	**CG**	**EG**
C14:0	5.59 ± 0.84	1.48 ^a^ ± 0.21	2.36 ^b^ ± 0.20
C16:0	27.02 ± 0.64	20.69 ^a^ ± 1.62	24.99 ^b^ ± 1.54
C18:0	5.88 ± 0.40	9.98 ^a^ ± 0.47	15.92 ^b^ ± 1.81
C18:1 cis-9	11.87 ± 0.96	20.53 ^a^ ± 0.51	17.04 ^b^ ± 0.12
C18:2 cis-9, cis-12	11.66 ± 0.52	37.08 ^a^ ± 0.84	25.73 ^b^ ± 0.34
C18:3 *cis*-9, *cis*-12, *cis*-15	37.98 ± 1.99	10.23 ^a^ ± 0.16	13.95 ^b^ ± 0.15

^1^ On a DM basis; ^2^ Data are reported as mean percentage of total fatty acids methyl esters (FAME) ± S.D. ^a,b^ Means with different superscripts are significantly different (*p* < 0.05).

**Table 2:** Chemical composition of individual milk obtained from the control group (CG) and from goats fed a dietary supplementation with olive leaves (EG).

Item	CG	EG	*p*
Casein, %	2.31 ± 0.33	2.29 ± 0.25	ns
Lactose, %	4.49 ± 0.17	4.41 ± 0.20	ns
Fat, %	2.61 ± 0.37	3.16 ± 0.46	ns
Protein, %	3.13 ± 0.37	3.11 ± 0.32	ns
Urea, mg/dL	38.60 ± 4.88	37.83 ± 4.75	ns
Milk production ¹ (mL)	1190 ± 45	1254 ± 50	ns

All data are reported as mean ± st. dev. ¹ Milk production for each animal for single milking. ns: not significant.

**Table 3:** Chemical composition and color evaluation of Caciotta Cheese at 1 (T1) and 60 (T60) d of ripening, obtained from goats fed a standard diet (CG) and goats fed a dietary supplementation of olive leaves (EG).

	T1			T60		
Item	CG	EG	*p*	CG	EG	*p*
Fat ¹, %	34.33 ± 1.25	33.37 ± 2.83	ns	33.36 ± 1.74	33.65 ± 1.99	ns
DM, %	56.79 ± 0.72	57.50 ± 0.42	ns	62.96 ± 0.61	65.02 ± 0.64	*
Color						
L*	58.13 ± 3.04	53.88 ± 2.97	**	44.79 ± 1.69	40.89 ± 1.41	**
a*	−2.33 ± 0.23	−2.47 ± 0.17	ns	−2.12 ± 0.05	−2.14 ± 0.08	ns
b*	3.47 ± 0.30	2.82 ± 0.23	*	2.10 ± 0.26	1.70 ± 0.14	**
YI	7.84 ± 0.85	7.51 ± 0.90	ns	6.37 ± 0.53	5.96 ± 0.51	ns
ΔE	4.30			3.92		

All data are reported as mean ± st. dev.; ¹ Data are reported on a dry matter (DM) basis; L*: lightness, a*: redness, b*: yellowness; * *p* < 0.05, ** *p* < 0.01, ns: not significant.

**Table 4:** Fatty acid profile of individual milk and fresh cheese (T1) obtained from goats fed a standard diet (CG) and a dietary supplementation of olive leaves (EG).

	Milk			Caciotta Cheese T1		
Fatty Acids ¹	CG	EG	*p*-Value	CG	EG	*p*-Value
C4:0	1.50 ± 0.53	1.39 ± 0.55	ns	1.40 ± 0.49	1.38 ± 0.29	ns
C6:0	1.99 ± 0.58	1.92 ± 0.58	ns	1.88 ± 0.51	1.88 ± 0.27	ns
C8:0	2.65 ± 0.69	2.64 ± 0.72	ns	2.54 ± 0.50	2.55 ± 0.33	ns
C10:0	10.14 ± 1.81	9.87 ± 2.30	ns	9.36 ± 1.28	9.21 ± 1.03	ns
C12:0	4.92 ± 0.89	4.52 ± 0.86	ns	4.28 ± 0.36	4.01 ± 0.31	ns
C14:0	11.99 ± 0.8	11.49 ± 1.14	ns	11.27 ± 0.33	11.17 ± 0.36	ns
C15:0	0.97 ± 0.10	0.96 ± 0.11	ns	0.89 ± 0.04	0.89 ± 0.01	ns
C16:0	28.14 ± 0.57	27.30 ± 0.35	*	26.49 ± 0.85	25.98 ± 0.48	ns
C17:0	0.61 ± 0.09	0.63 ± 0.08	ns	0.60 ± 0.03	0.61 ± 0.02	ns
C18:0	9.17 ± 0.49	10.34 ± 0.65	*	11.96 ± 0.88	12.20 ± 0.72	ns
C20:0	0.22 ± 0.04	0.26 ± 0.05	ns	0.22 ± 0.02	0.24 ± 0.03	ns
C22:0	0.07 ± 0.02	0.09 ± 0.02	ns	0.09 ± 0.05	0.08 ± 0.02	ns
SFA	72.35 ± 2.81	71.41 ± 3.42	ns	70.99 ± 1.66	70.17 ± 1.26	ns
C14:1 *cis*-9	0.44 ± 0.08	0.46 ± 0.08	ns	0.41 ± 0.02	0.43 ± 0.01	ns
C16:1 *cis*-9	0.31 ± 0.05	0.34 ± 0.05	ns	0.30 ± 0.01	0.33 ± 0.02	ns
C18:1 *trans*-11	0.38 ± 0.03	0.44 ± 0.02	*	0.56 ± 0.05	0.63 ± 0.07	*
C18:1 *cis*-9	18.62 ± 0.22	19.57 ± 0.34	**	20.13 ± 1.36	20.71 ± 1.01	ns
C18:1 *cis*-11	0.31 ± 0.06	0.31 ± 0.10	ns	0.23 ± 0.04	0.22 ± 0.03	ns
MUFA	20.05 ± 1.91	21.12 ± 2.04	ns	21.63 ± 1.37	22.32 ± 1.02	ns
C18:2 *cis*-9, *cis*-12	2.68 ± 0.39	2.43 ± 0.59	ns	2.43 ± 0.18	2.41 ± 0.12	ns
CLA	0.81 ± 0.54	0.98 ± 0.64	ns	1.08 ± 0.08	1.12 ± 0.03	ns
C18:3 *cis*-9, *cis*-12, *cis*-15	0.75 ± 0.03	0.85 ± 0.05	*	1.00 ± 0.06	1.13 ± 0.04	*
PUFA	4.25 ± 0.64	4.26 ± 0.65	ns	4.51 ± 0.33	4.66 ± 0.23	ns
Others	3.34 ± 0.44	3.21 ± 0.27	ns	2.87 ± 0.12	2.85 ± 0.03	ns
MUFA/SFA	0.28 ± 0.04	0.30 ± 0.06	ns	0.31 ± 0.03	0.32 ± 0.02	ns
PUFA/SFA	0.06 ± 0.01	0.06 ± 0.01	ns	0.06 ± 0.01	0.07 ± 0.01	ns
UFA/SFA	0.34 ± 0.05	0.36 ± 0.06	ns	0.37 ± 0.03	0.38 ± 0.02	ns
ω-6/ω-3	4.70 ± 0.26	4.00 ± 0.11	***	3.50 ± 0.07	3.12 ± 0.02	***
AI	3.39 ± 0.54	3.14 ± 0.64	ns	2.91 ± 0.22	2.77 ± 0.18	ns
TI	3.50 ± 0.52	3.30 ± 0.41	ns	3.14 ± 0.13	2.97 ± 0.09	ns
DI C14:1*cis*-9/(C14:1*cis*-9+C14:0)	0.04 ± 0.01	0.04 ± 0.01	ns	0.03 ± 0.01	0.04 ± 0.01	ns
DI C16:1*cis*-9/(C16:1*cis*-9+C16:0)	0.01 ± 0.01	0.01 ± 0.01	ns	0.01 ± 0.01	0.01 ± 0.01	ns
DI C18:1*cis*-9/(C18:1*cis*-9+C18:0)	0.67 ± 0.05	0.66 ± 0.04	ns	0.63 ± 0.01	0.63 ± 0.01	ns
DI CLA/(C18:1*trans*-11+CLA)	0.68 ± 0.08	0.67 ± 0.11	ns	0.66 ± 0.04	0.64 ± 0.02	ns
SCFA	3.49 ± 0.44	3.31 ± 0.34	ns	3.28 ± 0.99	3.25 ± 0.55	ns
MCFA	31.10 ± 2.53	29.94 ± 1.74	ns	28.75 ± 2.39	28.25 ± 2.03	ns
LCFA	62.07 ± 1.71	63.53 ± 1.98	ns	65.10 ± 3.34	65.65 ± 2.45	ns

¹ Data are reported as mean percentage of total FAME ± st. dev. SFA: Saturated Fatty Acids; MUFA: Monounsaturated Fatty Acids; PUFA: Polyunsaturated Fatty Acids; CLA: Rumenic acid; AI: Atherogenic index; TI: Thrombogenic index; DI: Desaturation index. SCFA: short chain fatty acids; MCFA: medium chain fatty acids; LCFA: long chain fatty acids. * *p* < 0.05; ** *p* < 0.01; *** *p* < 0.001; ns: not significant.

**Table 5:** Aromatic profile of Caciotta cheese at 1 (T1), 30 (T30), and 60 (T60) d of ripening, obtained from goats fed a standard diet (CG) and a dietary supplementation of olive leaves (EG).

	T1			T60		
VOC ¹	CG	EG	*p*-Value	CG	EG	*p*-Value
**Alcohols**	7.26 ± 0.07	9.31 ± 0.60	*	5.43 ± 0.68	4.61 ± 0.08	*
1-butanol, 3-methyl	nd	nd		0.92 ± 0.11	1.26 ± 0.10	ns
1-hexanol	0.76 ± 0.10	0.70 ± 0.05	ns	1.22 ± 0.26	0.94 ± 0.05	ns
1-hexanol, 2-ethyl	5.62 ± 0.46	7.56 ± 0.56	*	2.40 ± 0.25	1.03 ± 0.05	**
1-heptanol	0.5 ± 0.17	0.44 ± 0.02	ns	0.44 ± 0.05	0.28 ± 0.03	**
1-octanol	nd	nd		0.10 ± 0.03	0.11 ± 0.03	ns
1-nonanol	0.24 ± 0.09	0.42 ± 0.08	ns	0.21 ± 0.01	0.52 ± 0.03	***
2-nonanol	nd	nd		0.03 ± 0.01	0.30 ± 0.07	*
Benzyl alcohol	0.07 ± 0.03	0.09 ± 0.03	ns	0.04 ± 0.01	0.08 ± 0.02	ns
Phenylethyl alcohol	0.08 ± 0.01	0.10 ± 0.03	ns	0.07 ± 0.01	0.09 ± 0.04	ns
**Aldehydes**	11.87 ± 1.57	11.73 ± 0.86	ns	3.75 ± 0.65	4.18 ± 0.24	ns
Hexanal	7.13 ± 1.63	6.48 ± 0.46	ns	2.14 ± 0.48	2.92 ± 0.22	ns
Heptanal	0.71 ± 0.2	0.61 ± 0.06	ns	0.24 ± 0.03	0.45 ± 0.15	ns
2-heptenal	0.08 ± 0.02	0.02 ± 0.02	ns	nd	nd	
Octanal	0.8 ± 0.13	1.00 ± 0.05	ns	nd	nd	
2-octenal	0.05 ± 0.02	0.22 ± 0.10	*	0.15 ± 0.02	0.17 ± 0.04	ns
Nonanal	2.66 ± 0.20	3.04 ± 0.30	ns	0.27 ± 0.04	0.16 ± 0.03	*
2-nonenal	0.04 ± 0.04	0.03 ± 0.01	ns	nd	nd	
2-decenal	0.16 ± 0.04	0.16 ± 0.01	ns	0.19 ± 0.06	0.15 ± 0.02	ns
Benzaldehyde	0.14 ± 0.06	0.07 ± 0.01	ns	0.10 ± 0.03	0.04 ± 0.01	*
Benzaldehyde, 3-ethyl	0.09 ± 0.01	0.10 ± 0.05	ns	0.39 ± 0.11	0.19 ± 0.06	*
Phenylacetaldehyde	nd	nd		0.26 ± 0.09	0.10 ± 0.05	ns
**Carboxylic Acids**	72.30 ± 1.10	69.20 ± 3.19	ns	80.90 ± 2.43	55.02 ± 1.86	*
Butanoic acid	0.04 ± 0.02	0.05 ± 0.02	ns	1.55 ± 0.17	0.51 ± 0.03	*
Hexanoic acid	4.12 ± 0.26	3.66 ± 0.50	ns	13.32 ± 0.78	6.77 ± 0.62	*
Heptanoic acid	0.13 ± 0.01	0.13 ± 0.02	ns	0.12 ± 0.04	0.14 ± 0.04	ns
Octanoic acid	32.13 ± 2.87	26.39 ± 0.56	*	11.13 ± 0.58	6.67 ± 0.32	*
Nonanoic acid	nd	nd		0.08 ± 0.02	0.01 ± 0.01	**
Decanoic acid	35.79 ± 1.51	38.50 ± 2.42	ns	53.29 ± 3.09	41.07 ± 2.78	*
Dodecanoic acid	0.10 ± 0.02	0.46 ± 0.03	*	1.92 ± 0.08	0.01 ± 0.01	**
**Ethyl Esters**	0.04 ± 0.04	0.02 ± 0.01	ns	2.27 ± 0.11	17.46 ± 1.19	*
Hexanoic acid ethyl ester	nd	nd		0.66 ± 0.09	7.69 ± 0.67	*
Octanoic acid, ethyl ester	nd	nd		0.61 ± 0.04	6.18 ± 0.41	*
Decanoic acid, ethyl ester	nd	nd		0.88 ± 0.05	3.48 ± 0.29	*
Dodecanoic acid, ethyl ester	nd	nd		0.10 ± 0.01	0.10 ± 0.04	ns
Tetradecanoic acid, ethyl ester	0.04 ± 0.04	0.02 ± 0.01	ns	0.03±0.01	0.02 ± 0.01	ns
**Ketones**	6.52 ± 0.03	7.67 ± 1.68	ns	5.65 ± 0.25	17.35 ± 1.62	**
Acetoin	2.82 ± 0.12	1.42 ± 0.09	*	nd	nd	
2-heptanone	1.79 ± 0.08	2.15 ± 0.31	ns	2.42 ± 0.22	4.93 ± 0.43	*
2-nonanone	1.88 ± 0.06	4.07 ± 0.86	*	3.21 ± 0.21	12.41 ± 0.63	**
2-undecanone	0.03 ± 0.01	0.02 ± 0.02	ns	0.01 ± 0.01	0.01 ± 0.01	ns
**Lactones**	1.51 ± 0.08	1.08 ± 0.21	ns	1.84 ± 0.21	1.22 ± 0.18	*
δ-nonalactone	0.38 ± 0.15	0.30 ± 0.13	ns	0.05 ± 0.02	0.03 ± 0.02	ns
δ-decalactone	0.60 ± 0.09	0.41 ± 0.05	*	1.30 ± 0.10	0.87 ± 0.11	*
δ-dodecalactone	0.19 ± 0.02	0.16 ± 0.02	ns	0.25 ± 0.02	0.18 ± 0.01	*
δ-tetradecalactone	0.06 ± 0.01	0.05 ± 0.03	ns	0.10 ± 0.02	0.07 ± 0.01	ns
ε-dodecalactone	0.28 ± 0.03	0.16 ± 0.05	ns	0.13 ± 0.01	0.08 ± 0.04	ns
**Others**	0.50 ± 0.03	0.99 ± 0.18	*	0.15 ± 0.05	0.16 ± 0.04	ns
Ethylbenzene	0.05 ± 0.01	0.48 ± 0.04	***	0.08 ± 0.03	0.06 ± 0.03	ns
L-camphor	0.29 ± 0.06	0.29 ± 0.07	ns	nd	nd	
Caryophyllene	0.15 ± 0.01	0.23 ± 0.04	*	0.07 ± 0.02	0.10 ± 0.02	ns

¹ All data are reported as mean percentage of (VOCs) ± st. dev. nd: not detectable. * *p* < 0.05; ** *p* < 0.01; *** *p* < 0.001; ns: not significant.

**Table 6:** Densitometric analysis of SDS-PAGE protein bands (Figure 1) in fresh (T1) and 60-days ripened (T60) cheese samples obtained from Saanen goats fed the control diet (CG) and a diet supplemented with olive leaves (EG).

	T1			T60		
Protein ¹	CG	EG	*p-*Value	CG	EG	*p*-Value
αs2-CN	18.23 ± 1.34	17.79 ± 1.65	ns	10.16 ± 0.59	8.82 ± 0.86	ns
αs1-CN	15.77 ± 1.58	17.05 ± 1.37	ns	8.77 ± 0.64	8.25 ± 0.76	ns
β-CN	17.39 ± 0.99	17.22 ± 1.11	ns	11.23 ± 0.66	12.35 ± 1.01	ns
LMWP	48.60 ± 3.64	47.93 ± 1.87	ns	69.84 ± 1.22	70.58 ± 3.45	ns

¹ Data are reported as mean (%) ± S.D. of the total proteins found in the electrophoretic profile of each sample. α-CN: α-casein; β-CN: β-casein; LMWP: low molecular weight protein. ns: not significant.

**Table 7:** Texture profile analysis of cheeses at 1 and 60 days of ripening, obtained from goats fed a standard diet (CG) and a dietary supplementation of olive leaves (EG).

	T1			T60		
	CG	EG	*p*-Value	CG	EG	*p*-Value
Hardness (N)	20.16 ± 2.53	18.45 ± 1.36	ns	34.37 ± 3.37	50.36 ± 3.97	***
Cohesiveness	0.76 ± 0.03	0.73 ± 0.03	ns	0.70 ± 0.03	0.69 ± 0.02	ns
Gumminess (N)	15.44 ± 1.59	13.59 ± 1.25	ns	23.88 ± 2.16	34.89 ± 1.64	***
Young Module (N/mm^2^)	53.90 ± 5.16	50.15 ± 3.03	ns	70.33 ± 5.21	108.37 ± 8.59	***

All data are reported as mean ± st. dev. *** *p* < 0.001; ns: not significant.

**Table 8:** ANOVA of the results obtained from Quantitative Descriptive Analysis. Values with different superscripts in the same column are different at *p* < 0.05.

	Color	Eyes	Hardness	Elasticity	Solubility	Adhesiveness
CG	2.333 ^a^	2.833 ^a^	3.128 ^a^	1.917 ^a^	2.750 ^a^	1.333 ^a^
EG	3.083 ^a^	1.500 ^b^	3.002 ^a^	2.250 ^a^	2.250 ^a^	1.250 ^a^
Pr > F (Model)	0.077	0.001	0.219	0.435	0.146	0.818
Significant	No	Yes	No	No	No	No
